# Effects of a Community Health Worker-Led Multimedia Intervention on the Uptake of Cervical Cancer Screening among South Asian Women: A Pilot Randomized Controlled Trial

**DOI:** 10.3390/ijerph16173072

**Published:** 2019-08-23

**Authors:** Cho Lee Wong, Kai Chow Choi, Bernard M. H. Law, Dorothy N. S. Chan, Winnie K. W. So

**Affiliations:** The Nethersole School of Nursing, Faculty of Medicine, The Chinese University of Hong Kong, Hong Kong

**Keywords:** cervical cancer, cancer screening, community health worker, South Asians

## Abstract

The utilization rate of cervical cancer screening services among South Asian women is low. Multimedia interventions conducted by community health workers (CHWs) could potentially enhance the cervical cancer screening uptake among these individuals. This study aimed to investigate the feasibility and preliminary effects of a CHW-led multimedia intervention on cervical cancer screening uptake among this underprivileged group. This pilot study utilized a randomized wait-list controlled trial design. Forty-two South Asian women were recruited at six ethnic minority associations. Randomization of each organization into either the intervention arm or wait-list control arm was then performed. The intervention was conducted by the CHWs from the associations where the participants were recruited. Outcome measures were assessed and compared at baseline and immediately post-intervention. We demonstrated that the intervention was feasible as evidenced by the high consent rate and low withdrawal and attrition rates. The intervention arm showed a statistically significant improvement in perceived benefits (*p* = 0.001) and perceived barriers (*p* = 0.02). However, no significant difference was noted in screening uptake and screening intention between arms. Our findings support the feasibility of CHW-led multimedia intervention and provide preliminary evidence of its effectiveness on enhancing the cervical cancer screening beliefs among South Asian women.

## 1. Background

Cervical cancer is the fourth most common cancer among women worldwide; about 570,000 new cases were reported in 2018, with an age-standardized rate of mortality of 6.9 per 100,000 [1]. To address the high worldwide prevalence of cervical cancer, strategies need to be implemented to achieve effective prevention of this disease. Early detection of cancer via cancer screening is suggested to be an effective preventive strategy, as it enables earlier commencement of treatment, thus increasing the chance of successful treatment. Cancer screening was suggested to contribute to a lowering of the cancer mortality rate [2]. Currently, the Papanicolaou test (Pap test) is one of the most common and effective forms of cervical cancer screening [3]. The test has been made publicly available worldwide, and its regular utilization is promoted by the local government [4]. Notably, the Department of Health of Hong Kong recommends that women aged between 25 and 64 years old who have had a sexual experience undertake the Pap test every three years [4].

As the largest (30%) and one of the fastest growing ethnic minority groups in Hong Kong [5], it is important that South Asians (Indians, Nepalese, and Pakistanis) be subjected to health interventions for the enhancement of public health. Although most have settled in Hong Kong, and some were born and raised locally, they exhibit socioeconomic attributes that are distinct from those of other Hongkongers. For example, the education level of South Asians is relatively low, with only 38% of them having attained post-secondary education. Moreover, less than 10% of Pakistanis and Nepalese speak English or Cantonese in their daily conversations [5]. Most importantly, South Asian ethnic minorities have been found to have a lower utilization rate of cervical cancer screenings than individuals of the general population [6,7,8,9]. Our recent study demonstrated a significantly lower cervical cancer screening rate among South Asian ethnic minorities in Hong Kong compared with the general population, in which only 36.9% of South Asian women aged 18 or above were reported to have previously utilized the Pap test [9] compared with 60.5% of females in the general population of Hong Kong [10]. This phenomenon was suggested to be attributed to factors such as limited knowledge or misconceptions of cervical cancer and its prevention among South Asian ethnic minorities, a limited accessibility to cervical cancer screening services, cultural beliefs among South Asians, and a language barrier [6,9]. In view of these barriers, strategies need to be developed to enhance the knowledge of South Asian ethnic minorities on cervical cancer and its prevention. Any misconceptions that hamper these groups from undergoing cancer screenings should be clarified. Doing this could increase the intention of these individuals to undergo cancer screenings and, consequently, improve cancer screening uptake. One such strategy would be the delivery of health educational interventions aimed at increasing the awareness of South Asians of the importance of cancer screenings.

To date, a number of interventions that effectively promote cervical cancer screening intention have been reported and share common features, including being theory-based and culturally sensitive, usage of multiple educational strategies, within-community delivery, and provision of personnel for assistance in scheduling cancer screening appointments [11,12,13]. We intend to develop a culturally sensitive intervention that combines the use of multimedia and community health workers (CHWs) for enhancing cervical cancer screening intention among South Asian ethnic minorities within local communities in Hong Kong. This intervention is based on the features that have been identified to enhance the effectiveness of interventions that could potentially enhance cervical cancer screening uptake. The contents of the intervention are based on the health belief model, which is commonly used in the development of interventions aiming to modify health behaviors among intervention participants [14]. Specifically, the use of multimedia in interventions likely ensures that multiple educational strategies are employed for intervention delivery, thereby enhancing learning outcomes. As CHWs are individuals with the same ethnic origin as those within a local community, and share the same culture as their community peers and provide them with social support, they can help deliver interventions in a culturally sensitive manner. CHWs can also assist South Asian ethnic minorities in scheduling cancer screening appointments. Our previous study demonstrated the positive effect of a CHW training program in enhancing CHW competence in increasing community peer knowledge of cervical cancer and self-efficacy in cervical cancer screening utilization [15]. As training South Asian CHWs within the local community is feasible, we speculate that the innovative approach of combining multimedia and CHWs in intervention delivery can enhance knowledge of cervical cancer screening among participants. In addition, this approach can facilitate intervention participants in finding their way through health systems by tackling the barriers they encounter in making cancer screening appointments with healthcare providers. In this study, we intend to explore whether a multimedia, CHW-led intervention can be delivered within South Asian communities in Hong Kong. We also aim to examine whether this intervention can enhance cancer knowledge and screening intention among South Asian ethnic minorities in these communities.

## 2. Objectives

The objectives of this pilot trial were:

1. to assess the parameters on feasibility (screening, eligibility, consent, withdrawal rates during recruitment, and attrition rate) for conducting a CHW-led intervention among South Asians women;
2. to assess the preliminary effects of a CHW-led intervention for South Asian women compared with the controls on the (1) uptake rate of cervical cancer screening; (2) readiness to undergo cervical cancer screening; and (3) beliefs regarding cervical cancer screening immediately post-intervention.

## 3. Methods

### 3.1. Design

A pilot randomized wait-list controlled trial was conducted between September and December 2018. The trial was registered at the Chinese Clinical Trial Registry (ChiCTR1800017227) on 18 July 2018.

### 3.2. Study Settings

The trial was conducted in six non-governmental organizations that agreed to participate in this study. These organizations offer similar types of services, which are primarily health and translation services, to local South Asians. They also organize various religious and social activities and provide support services to local South Asians. 

### 3.3. Participants and Sample Size

Individuals who fit the following eligibility criteria were included in the trial: (1) women of a South Asian origin (Pakistani, Indian, or Nepali), (2) aged 25 or above, with previous sexual activity, (3) no previous cervical cancer screening in the past five years, (4) no previous cancer diagnosis, and (5) no educational intervention on cervical cancer screening in the past year. 

As suggested by Cocks and Torgerson [16], the sample size for a pilot trial should be about 10% of that required in the main trial. As the sample size of the main trial was 408 (as discussed in [17]), about 41 subjects were deemed sufficient. 

### 3.4. Recruitment, Randomization, and Blinding

The process of subject recruitment, intervention development, and outcome measures used are described in [17]. Briefly, six South Asian community centers or organizations located in various districts of Hong Kong were randomized into either the intervention arm or wait-list control arm. One CHW was recruited at each organization. The arm that each recruited CHW was allocated to was determined by the arm that their affiliated organization was randomized to. CHWs affiliated with organizations in the wait-list control arm delivered the intervention after post-intervention data collection.

To achieve blinding of the intervention deliverer, subject recruitment was carried out by a research assistant (RA) who was not involved in intervention delivery at the premises of the six South Asian organizations that participated in the study. The RA was a female South Asian who could communicate using South Asian languages. She was also a registered nurse with a Bachelor’s degree in nursing, who possessed considerable experience in subject recruitment for health promotion projects. The potential participants were screened for eligibility and recruited after they had provided their written informed consent. Cluster randomization was carried out by an independent statistician who generated a random allocation sequence using a computer-generated randomization scheme for allocation of the six organizations into either the intervention or wait-list control arms. The allocation of participants into either arm was based on the organization at which the participants were recruited at and which arm the organizations were allocated to. To ensure blinding of the data collection, another RA who was blinded to arm allocation was employed for this task. However, as the CHWs were involved in the intervention delivery, blinding of the intervention deliverers could not be achieved. 

### 3.5. Interventions

#### 3.5.1. The Intervention Arm

Within two weeks following the collection of the socio-demographics and baseline data upon subject recruitment, the CHWs directly contacted the participants to inform them of their schedule and venue for the intervention. The evidence-based multimedia educational intervention lasted for three months and was developed on the basis of the training program for CHWs developed by the project team [15] and recommendations from systematic reviews [11,12,13]. The intervention consisted of two parts: (1) a 30-min multimedia educational program presented using a structured PowerPoint slide with a video clip aiming to augment knowledge of cervical cancer and its prevention; (2) a monthly telephone follow-up once a month for three months and the provision of navigation assistance in accessing screening services. An information booklet containing information on cervical cancer was provided to each participant to recap what they had learned during the health talks. The content of the intervention is reported in [17].

#### 3.5.2. Control Arm

Participants allocated to the control arm received the intervention as described above after data collection at post-intervention. 

## 4. Outcome Measures

### 4.1. Feasibility of Conducting a Definitive Trial

#### Potential Participants That Screened

The number of potential participants approached and screened for eligibility by the RA.

### 4.2. Eligibility Rate

The number of participants fulfilling the inclusion criteria divided by the number of participants screened for the eligibility. 

### 4.3. Consent Rate

The number of participants who gave consent divided by the number of participants eligible for the study. 

### 4.4. Withdrawal Rate

The number of participants who withdrew from the study divided by the number of participants who provided the informed consent for the study. 

### 4.5. Attrition Rate

The number of participants who dropped out of the study before completion divided by the number of participants who provided informed consent for the study. 

## 5. Preliminary Effects of the Intervention

### 5.1. Uptake Rate of Cervical Cancer Screening

The primary outcome of this trial was cervical screening uptake. The finding was assessed by the receipt presented by the participants; the receipt proves that the participants have utilized local cancer screening services and that they have undertaken cervical cancer screening. 

### 5.2. Readiness to Undergo Screening

The participants’ perceived readiness to undergo cervical cancer screening was assessed via a single questionnaire item in which participants were asked to respond either “yes” or “no” to whether they would undertake a Pap test within the next month. 

### 5.3. Cervical Cancer Screening Beliefs

The cervical cancer screening belief scale is a 27-item instrument used to assess participants’ intentions to undergo cervical cancer screening. Participants rated each item using a five-point Likert scale. A higher score indicates a higher level of participants’ perception on the risk and severity of cervical cancer, as well as the benefits and barriers to cancer screening utilization and participants’ confidence in undergoing screening. The Nepali and Urdu versions of the instrument were generated through translation, and these versions were validated among a group of South Asian women in Hong Kong. The translated instruments demonstrated satisfactory reliability and validity [18,19].

### 5.4. Socio-Demographics and Cervical Cancer Screening Questionnaire

Socio-demographic information such as participants’ age, ethnicity, educational level, monthly household income, marital and employment status, and number of children were collected. Information related to family history of cervical cancer, possession of health insurance, and previous cancer screening utilization, were also obtained at baseline. 

#### 5.4.1. Data Collection

Upon receiving the participants’ informed consent, the RA, who was blinded to arm allocation, collected the data by measuring the primary and secondary outcomes via face-to-face interviews with a self-report questionnaire. For this pilot trial, the outcome variables were collected before the participants received the intervention (baseline: T0) and immediately after the intervention (T1). 

#### 5.4.2. Statistical Analysis

Data were presented using appropriate descriptive statistics. Independent *t*, chi-square, or Fisher’s exact tests were used to assess whether there were significant differences in the baseline characteristics between the intervention and control arms. The binary outcomes of ever having had a Pap test and willingness to undergo a Pap test in the next month were compared between the arms by chi-square or Fisher’s exact tests, as appropriate. The cervical cancer screening belief scores were compared between the two arms using independent *t*-tests. Statistical analyses were performed using SAS release 9.4 (SAS Institute Inc., Cary, NC, USA). All statistical tests were two-sided, and the level of significance was set at 0.05.

#### 5.4.3. Ethical Considerations

Ethical approval was obtained from the Research Ethics Committees of the authors’ study institutions prior to commencement of the research. The conduction of the study complied with the Declaration of Helsinki. Prior to the enrollment of eligible participants, our research team provided them with detailed information about the study’s aims and objectives in verbal and written format. The potential participants were provided adequate time to consider their decisions. All the participants were informed about their right to withdraw at any point without providing a reason once they had provided written informed consent. They were also assured that any data they provided during the study would be kept anonymous and confidential. Participants provided their informed consent before they participated in the study. 

## 6. Results

### 6.1. Feasibility of Conducting a Definitive Trial

**Figure 1:**
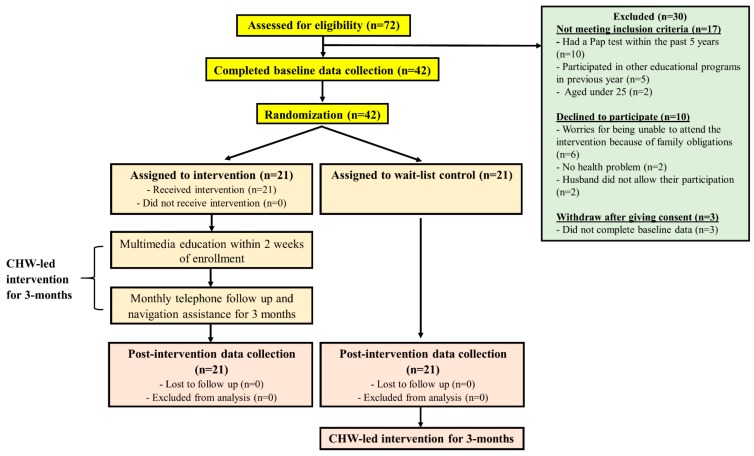
The flow diagram of intervention and data collection points. CHW: community health worker.

A total of 72 South Asian women were approached and screened for eligibility. Of these women, 17 failed to meet the inclusion criteria, mainly because they had received a Pap test within five years or had been exposed to another cervical cancer educational program last year. Interestingly, two participants who already had two children were approached by the RA during recruitment, but they were younger than 25 years old, resulting in an overall eligibility rate of 76%. Ten eligible participants refused to participate, mainly because they worried that they could not participate in the intervention due to family obligations (*n* = 6), showed no health problems prompting the need to participate (*n* = 2), or were prohibited by their husband from participating (*n* = 2). The final consent rate was 82%. Another three participants withdrew from the study after giving consent because they felt uncomfortable disclosing information related to cervical cancer (i.e., family history of cervical cancer or experience of having had a cervical disease before) during the baseline data assessment, resulting in a withdrawal rate of 7%. Accordingly, the pilot trial included 42 participants randomly allocated to either the CHW-led intervention (*n* = 21) or wait-list control arm (*n* = 21). Figure 1 shows the CONSORT diagram of the trial. All the participants in the intervention arm participated in the intervention. Meanwhile, all the participants in both arms completed the assessments at baseline and at T1, with an attrition rate of 0%. 

Table 1 shows the socio-demographic characteristics of the participants and Table 2 shows the health behaviors related to cervical cancer and screening. No statistically significant difference was observed between both arms except the numbers of children and those who received recommendations from friends or doctors.

### 6.2. Preliminary Effects of the CHW-Led Multimedia Intervention

Table 3 compares the primary and secondary outcomes between the control and intervention arms. Comparison of the changes in T0 and T1 between the two arms indicated that the intervention arm reported significant improvement in perceived benefits (*p* = 0.001) and reduction in perceived barriers (*p* = 0.02). An insignificant trend toward greater improvement was also noted in perceived susceptibility, perceived severity, and self-efficacy. However, no statistically significant difference was observed in either uptake of cervical cancer screening (*p* = 0.739) or screening intention (*p* = 0.999). 

## 7. Discussion

South Asian women are at a significant risk of developing cervical cancer, but their screening uptake rate is relatively low compared with that of the general population because of various factors such as attitudes, culture, or language that discourage them from undergoing screening [6,7]. This study provides novel evidence by supporting that combining the use of multimedia and CHWs in the delivery of educational interventions is an effective means for addressing the low cancer screening uptake among South Asian ethnic minorities. We employed an objective measurement—the presentation of a receipt as evidence for undergoing cancer screening—to evaluate the effectiveness of the intervention in enhancing cancer screening utilization among the participants. The pilot data suggests that the proposed intervention was highly feasible among our target population, providing a potentially effective strategy to increase cervical cancer screening uptake for this underprivileged group. 

This pilot first aimed to assess the feasibility of the study design. Previous studies have shown the challenge of recruiting South Asian women in research studies [20]. Nonetheless, our results revealed the feasible sample recruitment for this CHW-led multimedia intervention, as evidenced by an overall high consent rate and low withdrawal. In addition, comparable numbers of participants from three ethnicities were recruited, and all of them completed the three-month intervention. This finding may be due to several strategies that have been devised to improve subject recruitment and retention. In the initial stage, we encountered potential South Asian women who refused to take part because they were concerned about the intervention schedule and whether they would be able to attend due to family and childcare responsibilities. This condition is understandable, as South Asian women regard family obligations over other activities, even those that are beneficial to their health [20]. In addition, three participants refused to complete the baseline data after giving consent as they were reluctant to disclose sensitive issues such as family history of cervical cancer. To overcome these challenges, we alleviated their concerns by allowing participants to select the time of intervention to avoid intervening with their daily routine. A female South Asian RA collected the data in a private room and reinforced the confidentiality issues in the subsequent subject recruitment [19]. Additional meetings (from twice a week to once a week) were held with the RA to discuss the challenges encountered during subject recruitment and possible solutions. These strategies were proven to be effective and should therefore be considered in future studies to improve participation rates.

The second objective was to examine the preliminary effects of the CHW-led multimedia intervention. Although this pilot study lacked sufficient power to detect meaningful changes, and thereby insignificant results were expected, the changes in several screening belief outcomes in the intervention arm were encouraging. Compared with the control arm, intervention participants reported an improvement in perceived benefits and a reduction in perceived barriers toward cervical screening immediately after the three-month intervention. An insignificant trend toward greater improvement in perceived susceptibility and self-efficacy to undergo cervical cancer screening were also noted. These encouraging findings are likely attributed to the integration of multimedia and CHW approaches, minimizing barriers, and enhancing the self-efficacy of South Asian women to undergo cancer screening. The use of the multimedia approach would enable a more effective education of individuals with lower literacy levels, such as the South Asian ethnic minorities, in regards to what cervical cancer and its preventive measures are [21]. Moreover, the trained CHWs served as important resource persons impacting the health knowledge of their community peers. CHWs shared the same languages and possessed a good understanding of their cultural beliefs, which allowed them to fully understand the barriers that their peers encountered, thus providing them with appropriate support. In contrast, interventions reported by previous studies conducted in Western countries have primarily been delivered by health care professionals who were unlikely to share similar cultural backgrounds with the South Asian ethnic minorities. This difference in cultural background hinders the intervention deliverers from providing appropriate support based on participants’ cultural preferences [12]. The work of CHWs was further optimized by monthly phone calls to participants to enhance the knowledge they acquired during the intervention, and ultimately to translate their knowledge into changes in specific cervical screening beliefs. 

Nevertheless, the results showed that uptake rate and intention to undergo screening showed no difference in either arm. This finding likely occurred because this pilot research only assessed the outcomes immediately after the three-month intervention, due to practical considerations. Without a follow-up period, reflecting the changes in one’s health behavior, which generally requires a long period of time, may be difficult. One interesting point was that about 42.8% of the participants (57.1% in the control and 28.6% in the intervention) had already undergone a Pap test five years ago. The higher proportion of women in the control arm having undergone a Pap test is likely explained by the significantly higher number of those who received suggestions from friends or doctors to have the Pap test [6]. This result was unsurprising, as South Asian women show strong cultural cohesion, such that they usually follow recommendations of their peers. Meanwhile, all participants in the current study had given birth to children, and doctors in postnatal clinics often recommend women undergo cancer screening [20]. However, empirical evidence suggests that South Asian women underwent screening only to obey the recommendations of health care professionals without totally understanding the related reasons [6]. Without proper understanding of the importance of screening, the participants would likely fail to follow the screening schedule, which was the case for some of our participants that were screened more than five years ago. For instance, screening at an interval longer than three years is insufficiently effective for the prevention of cervical cancer, as pre-cancerous cells that are left untreated may change and turn into cervical cancer within 5–10 years [4]. 

Another interesting point was that, despite the majority of participants (100% in the control arm and 95.2% in the intervention arm) indicating that they would undergo a Pap test within the next month, the actual uptake rate disagreed with such willingness. Further studies are needed to investigate the reasons for this finding. Nevertheless, our preliminary findings suggest that an intervention dedicated to enhancing knowledge and targeting the screening beliefs of South Asian women is crucial, as changes in beliefs are an important factor contributing to changes in health behavior [14].

### Limitations

The current study features several limitations worthy of consideration. First, in view of the small sample size of this pilot trial, the outcome comparisons between the two arms were conducted without accounting for the potential design effect of the randomization conducted at a cluster level instead of individual participants. As we selected districts with similar types of services provided to local South Asians for cluster randomization, the number of clusters available for randomization was small (*n* = 6). This small number may have led to a bias owing to the potential heterogeneity in the characteristics of various clusters. Therefore, the preliminary effect estimates of the intervention on the outcomes should be interpreted with caution. The conduction of a future large-scale trial on the effectiveness of CHW-led multimedia intervention on cervical cancer screening uptake should involve the inclusion of a larger number of organizations available for cluster randomization. Second, given the nature of the intervention, blinding the participants and CHWs who carried out the intervention was impossible. Finally, no follow-up assessment was conducted on the screening uptake, and screening beliefs of South Asian women as a follow-up assessment at three months will be helpful in determining the long-term effects of the intervention. Despite these limitations, the outcome measures were assessed by the RA, who was blinded to arm allocation in order to reduce potential bias. The preliminary effects of the intervention were assessed by both subjective (self-reported questionnaires) and objective (receipt of screening utilization) measures. 

## 8. Conclusions

This pilot study suggests that the CHW-led multimedia intervention was a feasible and acceptable intervention for South Asian women. The preliminary results provided initial insights and evidence that multimedia intervention, together with a CHW approach, is crucial in enhancing cervical cancer screening beliefs among this underprivileged group. A full-scale study with a larger sample and longer follow-up period is warranted. 

## Figures and Tables

**Table 1:** Baseline characteristics of the participants (*n* = 42).

Characteristics	Control (*n* = 21)	Intervention (*n* = 21)	*p*-Value
Age (years) ^†^	41.57 (8.8)	41.43 (12.0)	0.965 ^a^
Ethnicity			
Pakistani	5 (23.8%)	9 (42.9%)	0.386 ^b^
Nepali	8 (38.1%)	7 (33.3%)	
Indian	8 (38.1%)	5 (23.8%)	
Educational level			
Primary school or below	4 (19.0%)	5 (23.8%)	0.723 ^c^
Secondary school	10 (47.6%)	7 (33.3%)	
College or above	7 (33.3%)	9 (42.9%)	
Have part-/full-time job			
No	13 (61.9%)	17 (81.0%)	0.172 ^b^
Yes	8 (38.1%)	4 (19.0%)	
Monthly family income (HK$)			
<10,000	2 (9.5%)	6 (28.6%)	0.079 ^c^
10,000–19,999	7 (33.3%)	10 (47.6%)	
≥20,000–29,999	9 (42.9%)	2 (9.5%)	
Don’t know	3 (14.3%)	3 (14.3%)	
Marital Status			
Separated/divorced/widowed	1 (4.8%)	1 (4.8%)	0.999 ^c^
Married	20 (95.2%)	20 (95.2%)	
Number of children			
1	5 (23.8%)	5 (23.8%)	0.005 ^b^
2	14 (66.7%)	5 (23.8%)	
≥3	2 (9.5%)	11 (52.4%)	

Variables marked with ^†^ are presented as mean (standard deviation), otherwise are frequency (%). ^a^ Independent *t*-test; ^b^ Chi-square test; ^c^ Fisher’s exact test.

**Table 2:** Health behaviors related to cervical cancer and screening.

Characteristics	Control (*n* = 21)	Intervention (*n* = 21)	*p*-Value
Family history of cervical cancer			
No	20 (95.2%)	17 (81.0%)	0.343 ^c^
Yes	0 (0.0%)	1 (4.8%)	
Don’t know	1 (4.8%)	3 (14.3%)	
Have had any cervical disease before			
No	20 (95.2%)	21 (100.0%)	0.999 ^c^
Yes	1 (4.8%)	0 (0.0%)	
Know the venue of clinics/centers providing cervical examinations			
No	12 (57.1%)	15 (71.4%)	0.334 ^b^
Yes	9 (42.9%)	6 (28.6%)	
Have had regular body check-up			
No	16 (76.2%)	15 (71.4%)	0.726 ^b^
Yes	5 (23.8%)	6 (28.6%)	
Have health insurance			
No	19 (90.5%)	17 (81.0%)	0.663 ^c^
Yes	2 (9.5%)	4 (19.0%)	
Any doctor suggested the participant have a Pap test			
No	14 (66.7%)	19 (90.5%)	0.130 ^c^
Yes	7 (33.3%)	2 (9.5%)	
Friends suggested the participant have a Pap test			
No	4 (19.0%)	12 (57.1%)	0.011 ^b^
Yes	17 (81.0%)	9 (42.9%)	
Family suggested the participant have a Pap test			
No	12 (57.1%)	17 (81.0%)	0.095 ^b^
Yes	9 (42.9%)	4 (19.0%)	
Ever received a reminder letter from doctor or healthcare organization for cervical examination			
No	16 (76.2%)	21 (100.0%)	0.048 ^c^
Yes	5 (23.8%)	0 (0.0%)	

^b^ Chi-square test; ^c^ Fisher’s exact test.

**Table 3:** Primary and secondary outcomes between control and intervention groups.

Outcomes	Control (*n* = 21)	Intervention (*n* = 21)	*p*-Value	Cohen’s d
***Screening behaviors***				
Ever had a Pap test (at least 5 years ago)				
T0	12 (57.1%)	6 (28.6%)	0.061	
T1	17 (81.0%)	12 (57.1%)	0.095	
Change from No at T0 to Yes at T1	6 (28.6%)	7 (33.3%)	0.739	
Willingness to undergo a Pap test within the next month				
T0	21 (100.0%)	20 (95.2%)	0.999 ^c^	
T1	21 (100.0%)	19 (90.5%)	0.488 ^c^	
Change from No at T0 to Yes at T1	0 (0.0%)	1 (4.8%)	0.999 ^c^	
***Cervical cancer screening belief***				
Perceived susceptibility ^†^				
T0	1.83 (0.95)	1.79 (0.83)	0.864 ^a^	
T1	1.67 (0.80)	1.98 (1.09)	0.299 ^a^	
Change (T1–T0)	−0.17 (0.98)	0.19 (0.84)	0.213 ^a^	0.39
Perceived severity ^†^				
T0	2.97 (0.83)	2.83 (0.94)	0.605 ^a^	
T1	3.03 (1.13)	3.56 (0.90)	0.104 ^a^	
Change (T1–T0)	0.06 (1.27)	0.73 (1.40)	0.114 ^a^	0.50
Perceived benefits ^†^				
T0	4.00 (0.51)	3.51 (0.59)	0.007 ^a^	
T1	4.12 (0.29)	4.25 (0.37)	0.231 ^a^	
Change (T1–T0)	0.12 (0.55)	0.73 (0.60)	0.001 ^a^	1.06
Perceived barriers ^†^				
T0	2.40 (0.67)	2.85 (0.82)	0.063 ^a^	
T1	2.26 (0.48)	2.07 (0.32)	0.147 ^a^	
Change (T1–T0)	−0.15 (0.82)	−0.78 (0.86)	0.020 ^a^	0.75
Self-efficacy ^†^				
T0	2.95 (1.14)	2.83 (1.32)	0.741 ^a^	
T1	3.49 (1.16)	4.21 (0.90)	0.032 ^a^	
Change (T1–T0)	0.54 (1.67)	1.38 (1.12)	0.062 ^a^	0.59

Variables marked with ^†^ are presented as mean (standard deviation), otherwise are frequency (%). ^a^ Independent *t*-test; ^c^ Fisher’s exact test.
